# Dietary Docosahexaenoic Acid and Glucose Systemic Metabolic Changes in the Mouse

**DOI:** 10.3390/nu15122679

**Published:** 2023-06-08

**Authors:** Bruce A. Watkins, John W. Newman, George A. Kuchel, Oliver Fiehn, Jeffrey Kim

**Affiliations:** 1 Department of Nutrition, University of California, Davis, Davis, CA 95616, USA; 2 Center on Aging, University of Connecticut Health Center, Farmington, CT 06030, USA; kuchel@uchc.edu; 3 United States Department of Agriculture, Agricultural Research Service, Western Human Nutrition Research Center, Davis, CA 95616, USA; john.newman2@usda.gov; 4 NIH UC Davis West Coast Metabolomics Center, Davis, CA 95616, USA; 5 Genome and Biomedical Sciences Facility, University of California, Davis, Davis, CA 95616, USA

**Keywords:** DHA, metabolomics, endocannabinoids, C57/blk6 mice, plasma, muscle, liver

## Abstract

The endocannabinoid system (ECS) participates in regulating whole body energy balance. Overactivation of the ECS has been associated with the negative consequence of obesity and type 2 diabetes. Since activators of the ECS rely on lipid-derived ligands, an investigation was conducted to determine whether dietary PUFA could influence the ECS to affect glucose clearance by measuring metabolites of macronutrient metabolism. C57/blk6 mice were fed a control or DHA-enriched semi-purified diet for a period of 112 d. Plasma, skeletal muscle, and liver were collected after 56 d and 112 d of feeding the diets for metabolomics analysis. Key findings characterized a shift in glucose metabolism and greater catabolism of fatty acids in mice fed the DHA diet. Glucose use and promotion of fatty acids as substrate were found based on levels of metabolic pathway intermediates and altered metabolic changes related to pathway flux with DHA feeding. Greater levels of DHA-derived glycerol lipids were found subsequently leading to the decrease of arachidonate-derived endocannabinoids (eCB). Levels of 1- and 2-arachidonylglcerol eCB in muscle and liver were lower in the DHA diet group compared to controls. These findings demonstrate that DHA feeding in mice alters macronutrient metabolism and may restore ECS tone by lowering arachidonic acid derived eCB.

## 1. Introduction

Numerous diet-related diseases stem from abnormal metabolic consequences. Examples include high glucose, non-esterified fatty acids, and acylethanolamide concentrations in blood of diabetes patients [1]. Elevated amino acid levels are also associated with diabetes [2]. Moreover, a recent metabolomic study showed altered levels of amino acids and increased oxylipin (OxL) inflammatory mediators in renal disease [3]. Thus, identifying changes in metabolite levels as a means of associating metabolites to a particular disease underscores the value of metabolomics analyses. Metabolomics is a science of systems biology that can broadly profile endogenous metabolites within a biological system, thus providing a snapshot of physiologic state or cellular status [4,5]. As such, metabolomics is well suited as a discovery tool for biomarker metabolites to accurately characterize a disease state to individual variability such as age, sex, genetics, diet, environmental conditions, and time of day [6,7].

Health status often reflects dietary habits, and diet modification is an attractive approach to prevent or control disease [8]. Several studies have demonstrated the beneficial effects of *n*-3 polyunsaturated fatty acids (PUFA) consumption in the prevention or treatment of diseases [9]. The endocannabinoid system (ECS) and its ligand endocannabinoids (eCB) are recognized as a homeostatic modulator of energy status specifically with respect to muscle [10] and systemic inflammation [11,12]. The ECS actions on maintaining homeostatic balance are linked to regulating food intake and directing energy metabolism at both the central and peripheral levels [13].

A review summarized findings on the effects of *n*-3 PUFA and glucose and fat metabolism in mice and cell cultures [14]. Some findings for glucose metabolism and physiology using molecular techniques in muscle and adipose tissues in the review are consistent with findings reported by Kim et. al. [10]. However, the review, which cites some studies of the authors, lacks a rigorous approach for experimental diet formulations and methods as those reported by Kim et al. in mice [10] and myoblasts [15].

An overactive ECS is believed to be one of the underlying causes of obesity, hyperglycemia, dyslipidemia, type 2 diabetes, and insulin resistance [5]. Accordingly, identifying ways to manage and normalize an overactive ECS, and the degree of stimulation or responsiveness of the system is dependent on the concentration of its ligands to activate its receptors involved in signaling [11]. Since the type of PUFA in the phospholipids of cellular membranes can change based on the type of dietary fat consumed, the downstream metabolic effects from alternations in the PUFA composition of membranes have a direct impact on the ECS [16,17]. Arachidonic acid (AA) is the precursor fatty acid for the biosynthesis of the two most studied endogenous ligand eCB, N-arachidonoyl ethanolamine (anandamide; AEA) and 2-arachidonoyl glycerol (2-AG), that activate cannabinoid receptors of the ECS [11,18].

In myoblast cultures, glucose use was observed to improve with docosahexaenoic acid (DHA) and the DHA-derived eCB, docosahexaenoyl ethanolamide (DHEA) treatments [15]. Moreover, when fed a DHA-rich diet, mice showed increased muscle mRNA and proteins involved in glucose uptake [10]. Thus, the focus of the current investigation was to distinguish shifts in metabolite changes in mice given a DHA-rich diet compared to the control diet. The overall research hypothesis is that DHA in a semi-purified diet when fed to C57/blk6 mice will restore endocannabinoid tone (action of ligands) and signaling of this system to improve macronutrient metabolism associated with reduced risk to obesity and diabetes.

The aim of this study was to investigate the effects of DHA intake on macronutrient metabolism in C57/blk6 mice from weaning to 112 d. Herein, plasma, skeletal muscle, and liver from C57/blk6 mice given a DHA semi-purified diet were analyzed for changes in metabolites by metabolomics analysis. Identifying metabolites provides insight to better understand the physiology within a biological system and a metabolic glimpse into the overall biological status. Thus, our hypothesis is that replacing tissue AA with DHA will change lipid and glucose metabolism and adipose accretion in mice. Our experimental design and hypothesis are a logical step to characterize the metabolite levels of macronutrients based on previous findings for DHA effects on glucose and fatty acid metabolism.

## 2. Materials and Methods

### 2.1. Mice and Semi-Purified Diets

Male C57/blk6J weanling mice (n = 80) were randomly assigned to two groups of 40 mice each and fed two customized AIN-93G diets (Dyets, Inc., Bethlehem, PA USA) that contained a DHA lipid or control lipid. The ingredient composition of the diets was published previously [10] and presented in Supplementary Table S1. Both diets contained the same amount of lipid, which was 11.04% of total diet by weight (25% energy). The control diet contained safflower oil only, while the treatment diet contained 6% of DHA as triglyceride (from DHASCO oil). All other ingredients were identical in the two diets. Thus, the two semi-purified diets were isocaloric and isonitrogenous, and had the same fat and protein levels to minimize ingredient composition impact on metabolism [19]. All mice were housed at 3 or 4 per cage in the University of Connecticut Health Center small animal facility. All mice were given diet and water ad libitum throughout the feeding period. *C57BL*/6J mice given free access to a high-fat diet develop obesity, mild to moderate hyperglycemia, and hyperinsulinemia [20]. The University of Connecticut Health Center has an Animal Welfare Assurance on file with the Office of Laboratory Animal Welfare (OLAW). The Assurance Number is A3471-01 and the effective dates are 27 April 2010–30 April 2014.

### 2.2. Collection of Samples and Body Mass Measurements

Body weight was recorded once a week for the duration of the study while food intake was recorded once a week for a month. Mice were subjected to dual energy X-ray absorptiometry (DXA) for body composition (pDEXA Densitometer Sabre; Norland, FL, USA) [21]. Diet was weighed at the time of administration and remaining food weighed at the end of 7 days. After 56 d of feeding, nine mice from the control and DHA groups were fasted for 8 h, weighed, and anesthetized with isoflurane. Blood was then collected for plasma using EDTA. The mice were euthanized by cervical dislocation. The gastrocnemius, fat pads, and liver, were weighed and immediately frozen in liquid nitrogen. The collection of tissue samples was repeated after 112 d of dietary treatment in both groups of mice. Mouse plasma, muscle (right gastrocnemius), and liver were subjected to metabolomics analyses.

### 2.3. Metabolomics Measurement in Mouse Plasma

Thirty µL mouse plasma samples were extracted with 1 mL −20 °C cold water/acetonitrile/isopropanol mixture (2/3/3, *v*/*v*/*v*). Dried extracts were derivatized by methoximation and trimethylsilylation for primary metabolite analyses [22]. A 0.5 µL volume was injected in glass liners without glass wool with 25 s splitless time into an Agilent 6890 gas chromatograph equipped with a 30 m long, 0.25 mm internal diameter Rtx-5Sil MS column with 0.25 mm 95% dimethyl/5% diphenyl polysiloxane film and an additional 10 m integrated guard column. Helium of 99.9999% purity with built-in purifier was used at a constant flow of 1 ml per min. The oven temperature was held constant at 50 °C for 1 min, and then ramped at 20 °C per min to 330 °C and held constant for 5 min. Mass spectrometry was performed on a Leco Pegasus IV time-off light mass spectrometer controlled using Leco ChromaTOF software version 4.1. For sample introduction, the transfer line temperature between gas chromatograph and mass spectrometer was set to 280 °C; 70 V electron ionization was employed, with an ion source temperature of 250 °C. Data acquisitions were performed after 290 s solvent delay, and mass spectra were acquired at mass resolving power R = 600 from *m*/*z* 85–500 at 17 spectra per second and 1850 V detector voltage without turning on the mass defect option. Recording ended after 1200 s. The instrument performed auto-tuning for mass calibration using FC43 (perfluorotributylamine) before starting analysis sequences.

### 2.4. Metabolomic Measurements in Mouse Muscle and Liver

Non-targeted metabolic profiling was conducted on 9 muscle and liver samples from each group of mice (at 56 d and 112 d of feeding, control and DHA dietary groups) using three independent platforms: ultrahigh performance liquid chromatography/tandem mass spectrometry (LC/MS) optimized for basic species, LC/MS optimized for acidic species, and gas chromatography/mass spectrometry as described previously [23,24]. Metabolites were identified by automated matching to chemical reference library standards based on retention time, molecular weight (*m*/*z*), preferred adducts, and in-source fragments as well as associated MS spectra, and were curated using software developed at Metabolon, Inc. (Morrisville, NC, USA) [25]. Following log transformation and imputation with minimum observed values for each compound, Welch’s two-sample t-test (statistical significance at *p* ≤ 0.05) was used to identify analytes that differed significantly between experimental groups.

### 2.5. Targeted Lipid Mediator Measurements 

Endocannabinoids and oxylipins were isolated from 250 µL of plasma using solid phase extraction and quantified by liquid chromatography tandem mass spectrometry as previously described [1]. Briefly, plasma was thawed on ice, and mixed with deuterated endocannabinoid and oxylipin internal standards in the head space of 60 mg Oasis-HLB solid phase extraction column (Waters Corp., Milford, MA, USA), where they were up-diluted to 20% methanol/0.1% acetic acid, and gravity loaded onto the columns, followed by vacuum to remove solvent. The columns were then wetted with 0.2 mL methanol and gravity eluted with 1.5 mL ethyl acetate. Solvent was removed by vacuum and residues were reconstituted in 50 µL methanol containing the internal standard 1-cyclohexyl-3-dodecyl-urea (Sigma, Aldrich, St. Louis, MO, USA). The resulting samples were filtered and analyzed by UPLC-(ESI)MS/MS by back-to-back (+)-mode/(−)-mode injections for endocannabinoid and oxylipin levels, respectively.

### 2.6. Tissue Gene Expression

The mRNA for gene expression was measured to understand changes in the ECS and glucose-related genes and their associated proteins (CB1, CB2, GLUT4, and insulin-R). All results from quadriceps and epididymal adipose tissue were previously reported [10], but these values were used in the analysis of all metabolomics data for metabolites measured in the present study. The section that follows, Section 2.7 Statistical analyses, describes the partial least squares discriminate analysis for all gene and metabolite data. For the gene measurements in brief, tissues were washed in PBS and homogenized in TRIzol (Invitrogen Corp., Carlsbad, CA, USA) and treated with DNase I (Ambion, Carlsbad, CA, USA) to remove DNA and isolate pure RNA. cDNA was synthesized from RNA (1 µg) using RNA transcriptase superscript III (Invitrogen Corp., Carlsbad, CA, USA), and used for quantitative RT-PCR with previously reported primer sequences [10]. All samples were analyzed in triplicate. Fluorescence emission was detected, and cycle threshold (CT) values were calculated in the linear range automatically. Relative CT amounts were calculated from the standard curve for each gene, which were normalized to GAPDH expression afterwards.

### 2.7. Statistical Analyses

Mouse plasma metabolomics data included 134 named compounds and 396 unknown compounds within treatment groups (control and DHA) that are presented as means ± SD in tables. Differences between dietary groups in tables were analyzed for significance by Student *t*-test (SAS for Windows version 9.3, SAS Institute Inc., Cary, NC, USA). Significance level was defined as *p* < 0.05. For metabolites in mouse liver and muscle samples, two types of statistical analysis were performed: (1) significance tests and (2) classification analysis. Pairwise comparisons were performed by Welch’s *t*-tests and/or Wilcoxon’s rank sum tests. For other statistical analyses, repeated measures ANOVA were done. Random forest analyses were done for classifications. Random forests give an estimate of how well we can classify individuals in a new data set into each group, in contrast to a univariate test that studies whether the unknown means for two populations are or are not different. Metabolic pathways in which a named compound was a potential intermediate were constructed using Microsoft Powerpoint (Microsoft Corporation, Redmond, WA, USA).

Random forests create a set of classification trees based on continual sampling of the experimental units and compounds. Then, each observation is classified based on the majority votes from all the classification trees. Statistical analyses are performed with the program “R” http://cran.r-project.org/ (accessed on 1 January 2019).

Descriptive statistics were calculated to inspect the distributional properties of all metabolites, mRNA, and proteins for gene expression in plasma, liver, muscle, and epidydimal fat pad. All analyses were conducted using R statistical software version 4.0.5 [26]. Wilcoxon signed-rank tests were performed to examine the changes between 56 d and 112 d. Statistical significance was determined at 0.05 alpha level and an effect size was computed for each comparison. Partial least squares discriminate analysis (PLS-DA) performed using leave one out cross validation was used to visualize metabolite differences in mice at both time points. Analyses were performed using auto-scaled data after the imputation of metabolites missing when at least 66% complete [3]. All metabolites were used to perform the analysis. Metabolites with variable importance in projections (VIP) > 1 were considered significant in group discrimination. Metabolites with VIP > 1 were combined with gene expression data and subjected to a hierarchical cluster analysis using a Ward agglomeration. 

## 3. Results

### 3.1. Mouse Food Intake, Body Weights, and Fat and Lean Mass

Food intake did not vary between the DHA diet and control groups over 112 d. Body weights were higher in the control group compared to the DHA diet group at 56 d (31.1 ± 3.2 and 28.1 ± 1.7 g) and 112 d (39.7 ± 4.0 and 35.7 ± 3.8 g), respectively, as shown in Supplementary Table S2. No difference was found between the two groups for lean and fat mass at 56 d, however, the control had higher fat mass and lower lean mass at 112 d (*p* = 0.005). The fat mass 0.59 ± 0.14 and 0.40 ± 0.11, and lean mass 0.32 ± 0.13 and 0.50 ± 0.10, in g/g body mass for control and DHA diet groups, respectively, were significant at 112 d as shown in Supplementary Table S2.

### 3.2. Primary Metabolite Levels in Plasma of Mice Fed the Semi-Purified Diets

Mouse plasma samples were analyzed for untargeted metabolite determination on a GCTOF MS platform with a total of 530 compounds identified. Among the named metabolites identified, 61 differed by *t*-test between the DHA and control groups, with 39 higher in the DHA group. Changes in amino acid metabolism of both essential and nonessential amino acids were detected (Table 1). Of the nine amino acids (essential: tryptophan, threonine, and phenylalanine; nonessential: tyrosine, serine, proline, glycine, alanine, and aspartic acid) that differed between the DHA diet group and control group, seven were lower in the DHA group, while two (phenylalanine and glycine) were higher (Table 2 and Supplementary Figure S1). A graphic representation of changes in the metabolism of amino acids is shown in Supplementary Figures S1 and S2, with emphasis on catabolism of amino acids in Supplementary Figure S2.

Changes in plasma fatty acids were also found between the DHA diet and control group. For example, lauric acid, palmitic acid, palmitoleic acid, oleic acid, linoleic acid, and eicosenoic acid were higher in the DHA group plasma (Table 2). Lower levels of arachidic acid and AA were found in the DHA group, which in part reflect the dietary treatment effects (Table 2). With respect to carbohydrate levels in the plasma, maltose, levanbiose, and fructose were lower in the DHA group, while fucose was higher in the DHA group (Table 2). Metabolites of fatty acid catabolism and glucose use associated with the Krebs cycle and glycolytic pathways, respectively, are shown in Supplementary Figure S3. The metabolic intermediates, pyruvic acid, malic acid, lactic acid, and alpha-glycerol phosphate (αGP) were lower in the DHA group (Table 3 and Supplementary Figures S1 and S3).

Glucuronic acid, ethanolamine, 3-hydroxy-3-methylglutaric acid, 2-hydroxybutanoic acid, 3-hydroxybutanoic acid, and adenosine-5-phosphate were higher in the DHA group (Table 4 and Supplementary Figures S3 and S4). Higher amounts of adenosine were found in the DHA group of mice compared to the control group (Table 4).

The nucleotides uridine, uracil, thymine, thymidine, adenosine, and adenosine-5-phosphate were higher in the DHA group, which may indicate accelerated metabolism of nucleotides (Table 4 and Supplementary Figure S5). The level of alpha-tocopherol was lower in the DHA group (Table 4). Pyrophosphate was the only inorganic compound found from the analysis and was higher in the DHA group (Table 4 and Supplementary Figure S2).

### 3.3. Metabolite Measurements in Mouse Muscle and Liver

#### 3.3.1. Levels of *n*-3 PUFA and Glycerolipids in Mice Fed the Semi-Purified Diets

After 56 d of feeding, the levels of DHA and other *n*-3 PUFA were increased in both liver and muscle in mice fed the DHA diet compared to the controls, showing a reciprocal effect on *n*-3 and *n*-6 PUFA in these tissues. For example, levels of DHA, EPA, and *n*-3 DPA were all higher in liver and muscle; while AA and *n*-6 DPA were lower in mice fed the DHA diet compared to the control group (Table 5). The analysis of all tissues from mice showed that feeding DHA led to higher tissue *n*-3 PUFA levels, with a corresponding decrease in AA and *n*-6 DPA levels. This is also true for the total *n*-3 PUFA and total *n*-6 PUFA. The lower levels of *n*-6 PUFA and higher *n*-3 PUFA levels in mouse muscle and liver tissues are consistent with the feeding of the DHA diet [10,15]. The changes in the levels of precursor PUFA for the synthesis of eCB provide a firm foundation for the rationale to investigate dietary interventions on changes of the ECS ligands. After 112 d of feeding the semi-purified diets, the DHA diet resulted in higher DHA, eicosapentaenoic acid (EPA), and *n*-3 DPA whereas AA, adrenate (22:4*n*-6), and *n*-6 DPA were decreased in liver and muscle of mice fed a DHA diet compared to the controls (Table 5).

At 56 d of feeding, DHA samples were found to be enriched in complex lipids (such as triglycerides and phospholipids) at the expense of *n*-6 PUFA as observed in monoacylglycerols and lysophospholipids (Table 6). The 2-DHA-GPE level was higher in both liver and muscle. The 1-docosahexaenoylglycerol (1-mono DHA) amount was higher in the muscle tissue of DHA-fed mice. In contrast to DHA enrichment in the complex lipids, the incorporation of AA and *n*-6 DPA into lysolipids (including those with choline, ethanolamine, and inositol headgroups) and monoacylglycerols was lower both in the liver and the muscle tissues in mice given the DHA diet compared to the controls.

After 112 d of being fed the semi-purified diets, higher levels of the complex lipids 2-DHA-GPE and 1-mono-DHA were found in both liver and muscle of DHA-fed mice compared to the controls (Table 6). In contrast, the levels of 1-AA-GPE, 2-DPA-GPE, 1-AA-GPC, and 2-DPA-GPC were lower in mice given the DHA diet (Table 6). Feeding the DHA semi-purified diet also led to changes in the levels of several core intermediates in liver complex lipid synthesis, such as glycerol (lower in the DHA group), ethanolamine (higher in the DHA group), 3-phosphoglycerate (3-PG, lower in the DHA group), glycerol-3-phosphate (lower in the DHA group), and CDP-choline (higher in the DHA group).

#### 3.3.2. Levels of Major Endocannabinoids and Endocannabinoid-like Compounds in Mice Fed the Semi-Purified Diets

After 56 d of diet treatment, the level of the endocannabinoid-like compound palmitoyl ethanolamide (PEA) was higher in the liver and muscle of mice fed the DHA diet (Table 7). The level of oleic ethanolamide was also higher in muscle with DHA diet feeding (Table 7). The N-palmitoyl taurine and oleoyltaurine were also higher in the liver in the DHA group compared to the control (Table 7). These results suggest that consumption of DHA led to an increased synthesis or decreased breakdown of endocannabinoid-like compounds. However, a lower level of 1-AG and 2-AG in the muscle at 56 d and 112 d in mice fed the DHA diet demonstrates the effects of DHA on AA-derived eCB (Table 7).

#### 3.3.3. Levels of Fatty Acid Oxidation and Catabolism Products of Mice Fed the Semi-Purified Diets

Muscle tissue from DHA-fed mice had a higher energy demand as evidenced by the higher level of ADP and lower AMP (Table 8). After 112 d of being fed the semi-purified diets, the acyl-carnitines were not different between the mice in the DHA group and the controls (Table 8). Among the compounds found significant was 3-hydroxybutyrate in liver that was sustained in the DHA group at 112 d (Table 8). A high level of fatty acid oxidation was observed in mouse muscle as acyl-carnitines (e.g., palmitoylcarnitine, oleoylcarnitine) and several acyl-glycines (e.g., hexanoylglycine and 3-hydroxybutyrate) were higher in the gastrocnemius muscle of DHA-fed mice (Table 9).

#### 3.3.4. Levels of Glucose and Glycolytic Intermediates in Muscle and Liver of Mice Fed the Semi-Purified Diets

Levels of metabolites in mice fed the DHA semi-purified diet suggest a higher use or greater muscle oxidation of glucose compared to the control mice at 112 d. For example, metabolites associated with glycolysis (e.g., pyruvate, 3-phosphoglycerate, and phosphoenolpyruvate) were significantly lower in mice fed the DHA diet compared to the controls (Table 9). This change in glucose levels was observed in both the muscle and liver tissues. Pyruvate-derived lactate was also lower in liver and muscle of the DHA-fed mice (Table 9). The sole exception to the lower level of intermediates was a significantly higher level of 2-phosphoglycerate in the liver (Table 9). Maltose and its maltooligosaccharides were lower in both the liver and muscle of the mice fed the DHA diet (Table 9). Lastly, lower levels of ribulose and ribose-5-phosphate in the liver indicate that the pentose phosphate pathway was lower in liver of the mice given the DHA diet compared to the controls (Table 9). Hence, reduced dependency on glucose catabolism in these tissues, but a greater flux through pathways of fatty acid oxidation occurs in mice fed the DHA diet.

After 112 d of being fed the semi-purified diets, the liver glucose levels were lower in DHA-fed mice compared to the control group, indicating that the feeding of a DHA diet modified liver metabolism of macronutrients via pathways of glucose metabolism (Table 9). Similarly, lower levels of fructose, lactate, pyruvate, maltose, and maltooligosaccharides were observed in mice fed the DHA diet compared to the controls (Table 9). Ribose was lower in the liver of mice given the DHA diet, indicating a possible lower activity of the pentose phosphate pathway (Table 9) and need for fatty acid synthesis.

#### 3.3.5. Levels of Additional Metabolites in Liver and Muscle for Mice Fed the Semi-Purified Diets

At 56 d of feeding, levels of glutathione were lower in the DHA group for both liver (0.15 ± 0.12 in the DHA group versus 2.04 ± 0.35 in the control, *p* < 0.0001) and muscle (0.42 ± 0.24 in the DHA group versus 1.5 ± 0.45 in the control, *p* = 0.0001) compared to the control and consistent with lower tocopherol. 2-hydroxybutyrate (2.93 ± 2.05 in DHA group compared to 0.52 ± 0.17 in control, *p* = 0.0010) was higher in the muscle of mice fed the DHA diet. Coenzyme A (0.10 ± 0.07 in DHA group versus 1.19 ± 0.209 in control, *p* < 0.0001) and 3-dephosphocoenzyme A (0.14 ± 0.11 in the DHA group versus 2.86 ± 0.78 in the control, *p* < 0.0001) were lower in liver of mice fed the DHA diet. However, pantothenic acid (1.77 ± 0.51 in the DHA group and 0.44 ± 0.14 in the control, *p* < 0.0001), the starting material for coenzyme A biosynthesis, was elevated in the liver of mice given the DHA diet. After 112 d of being fed the semi-purified diets, glutathione (GSH) was lower in both liver and muscle of DHA-fed mice (1.69 ± 0.36 in control and 0.94 ± 0.11 in DHA group, *p* < 0.0001).

Sulfur-containing amino acids levels in liver varied between the groups of mice: methionine (0.77 ± 0.09 in the control and 1.05 ± 0.10 in the DHA group, *p* = 0.0046), cysteine (1.36 ± 1.16 in the control and 0.75 ± 0.50 in the DHA group, *p* = 0.053), hypotaurine (0.865 ± 0.577 in the control and 1.286 ± 0.41 in the DHA group, *p* = 0.0454), and taurine (0.938 ± 0.18 in the control and 1.16 ± 0.19 in the DHA group, *p* = 0.041). Total AMP (2.51 ± 1.37 in the control and 0.99 ± 0.05 in the DHA group, *p* = 0.0229), guanosine (1.64 ± 0.45 in the control and 1.02 ± 0.13 in the DHA group, *p* = 0.001), and adenine (1.79 ± 0.28 in the control and 0.87 ± 0.12 in the DHA group, *p* < 0.0001) were different as well. The lower levels of these compounds in the livers of DHA-fed mice might suggest that purine oxidation was altered following DHA dietary treatment at 112 d. 

The downstream catabolites in pathways for hypoxanthine (0.70 ± 0.134 in the control and 1.29 ± 0.13 in the DHA group, *p* < 0.0001), xanthine (0.728 ± 0.070 in the control and 1.03 ± 0.05 in the DHA group, *p* < 0.0001), and urate (0.50 ± 0.25 in the control and 1.37 ± 0.86 in the DHA group, *p* = 0.0090) were higher in the liver of mice given DHA. Coenzyme A (1.51 ± 0.47 in the control and 0.92 ± 0.35 in the DHA group, *p* = 0.0132) was lower in liver of the DHA-fed mice at 112 d of being fed the semi-purified diets. However, pantothenate (0.50 ± 0.07 in the control and 1.37 ± 0.33 in the DHA group, *p* < 0.0001), and phosphopantetheine (0.92 ± 0.16 in the control and 2.17 ± 0.37 in the DHA group, *p* < 0.0001) were higher in liver of mice fed DHA compared to the controls. The differences in coenzyme A and pantothenate in the mice fed the DHA diet compared to the levels in the mice fed the control diet may support the finding of greater fatty acid oxidation.

### 3.4. Partial Least Squares Discriminant Analysis

**Figure 1:**
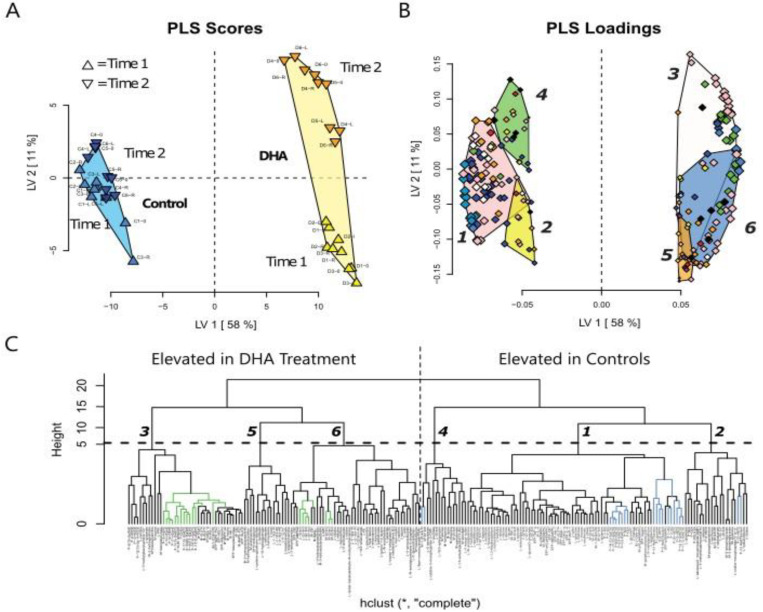
Panels (**A**–**C**). Mouse DHA feeding study Multivariate Analysis of measurements as PLS Scores, PLS Loadings, and hierarchical cluster analyses dendrogram. Partial Least Squares Discriminant analysis was performed using leave one out cross validation. PLS scores and PLS loadings. Of the 651 variables analyzed, 211 showed variable importance in projections (VIP) Scores > 1. These variables were isolated and subjected to a second PLS-DA analysis and the results are shown below. A hierarchical cluster analysis was performed on the correlation matrix of these variables and the cluster dendrogram was pruned to describe six data clusters, of which 3, 5, and 6 were higher in the DHA group and 4, 1, and 2 higher in the control group. On the following pages, the clusters are individually displayed, with VIP scores indicated for each variable at >1.75 (***), >1.5 (**), >1.25 (*), and >1 (◌). DHA = Mice fed the DHA diet.

Figure 1A,B shows the results of the Partial Least Squares Discriminant Analysis (PLS-DA) for all data using leave one out cross validation for PLS scores and PLS loadings. Figure 1C is the hierarchical cluster analyses dendrogram for six data clusters individually displayed with VIP scores indicated for each variable at >1.75 (***), >1.5 (**), >1.25 (*), and >1 (◌). The clusters 3, 5, and 6 are values higher in the DHA group compared to the control group. Clusters 4, 1, and 2 are values higher in the control group.

**Figure 2:**
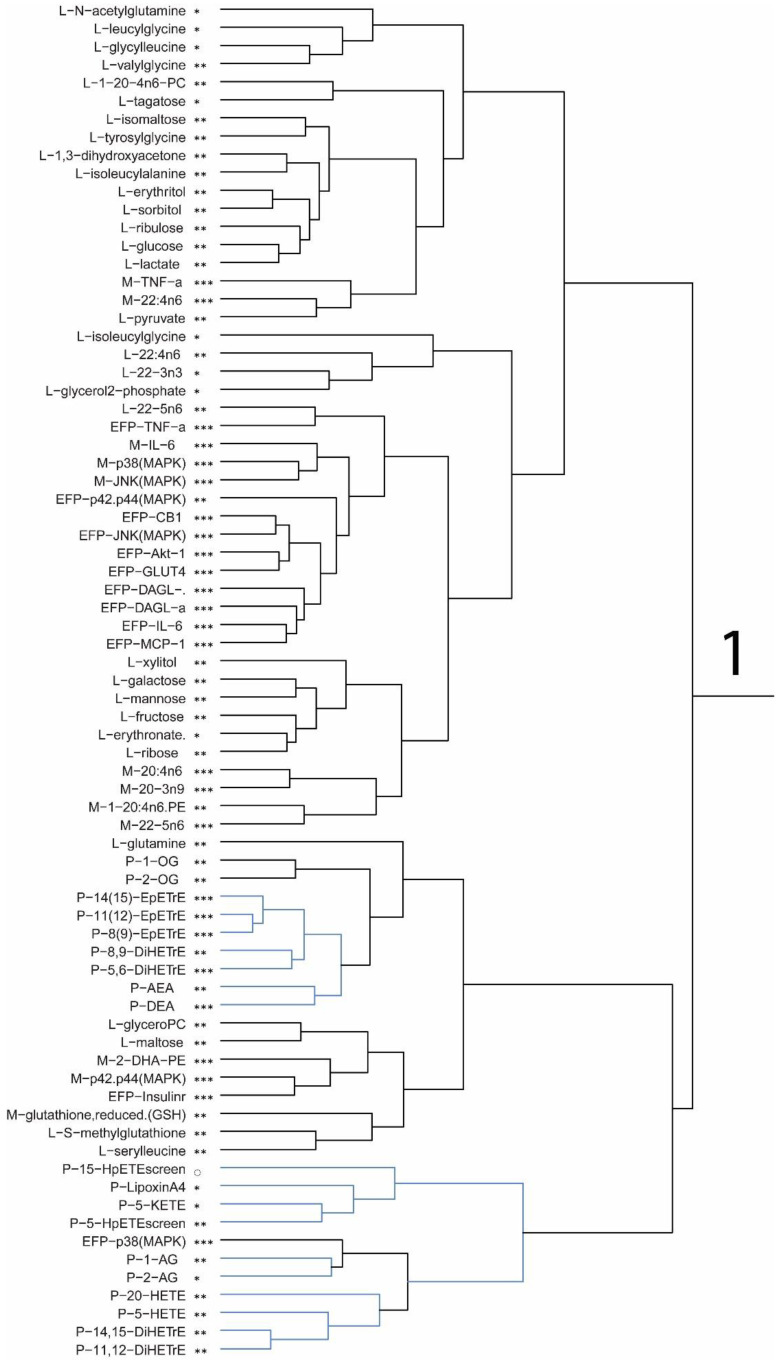

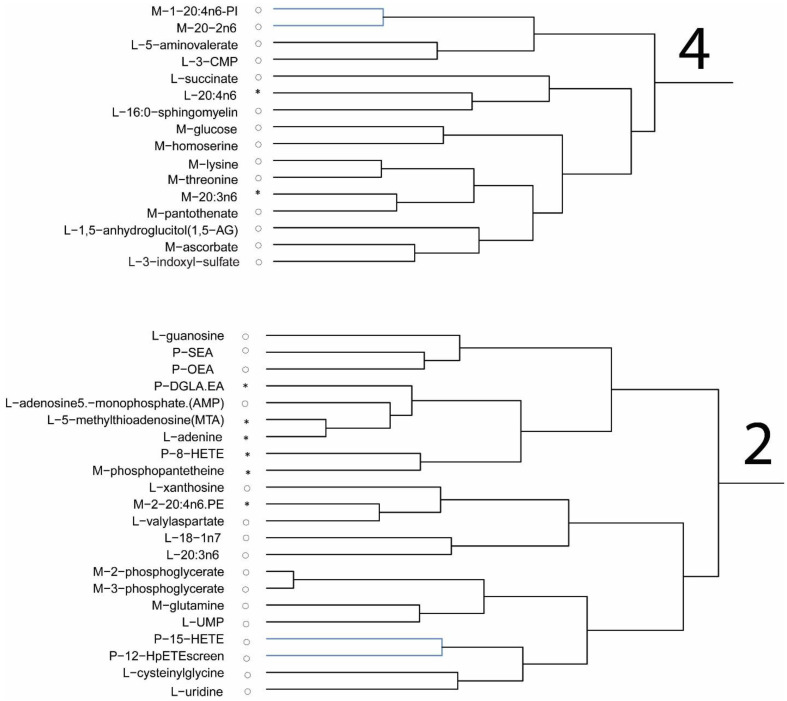

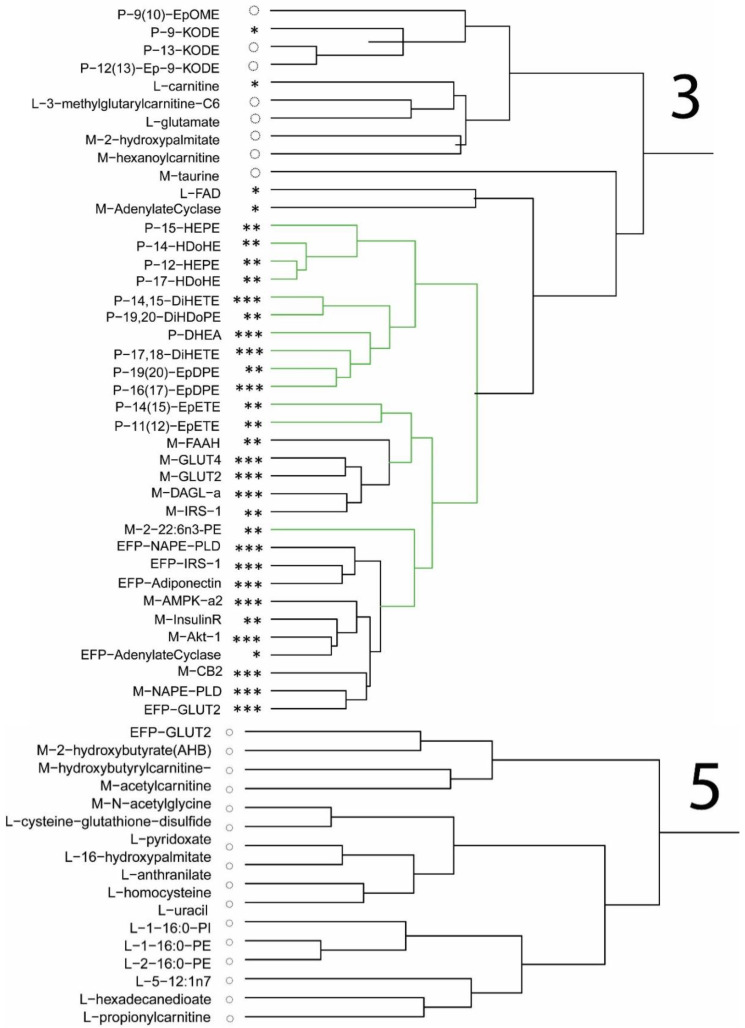

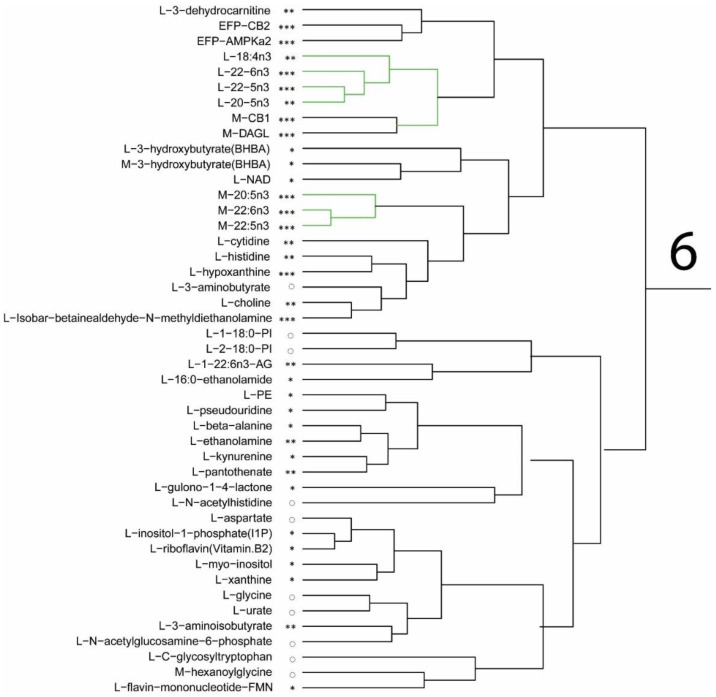
The hierarchical cluster analyses dendrogram are presented as six data clusters individually displayed with VIP scores indicated for each variable at >1.75 (***), >1.5 (**), >1.25 (*), and >1 (◌). Values for compounds with the preceding letters P, L, and M refer to plasma, liver, and muscle, respectively. EFP is epididymal fat pad qPCR values.

Figure 2 presents an enlarged illustration of the six clusters of metabolite values and the results of published values for gene products, mRNA, and protein. 

Values for compounds with the preceding letters P, L, and M refer to plasma, liver, and muscle, respectively. In addition, EFP is gene expression for epididymal fat pad qPCR values. Of the 651 variables analyzed, 211 showed variable importance in projections (VIP) Scores >1. The hierarchical cluster analysis was performed on the correlation matrix of these variables and the cluster dendrogram was pruned to describe six data clusters. The clusters are individually displayed, with VIP scores indicated for each variable at >1.75 (***), >1.5 (**), >1.25 (*), and >1 (◌). The dietary treatment DHA for mice fed the DHA diet and mice fed the control diet. The data shown reflect the metabolites measured in P, L, and M herein and included is a new data analysis of gene products, plasma eCB, and OxL measured in the mice from our laboratory [10]. The application of new data analysis with the metabolite levels in the current study helps to show the relationships between metabolites and molecular factors that influence macronutrient metabolism in mice.

The PLS scores in Figure 1 illustrate the relationships for all measurements in the control and DHA groups for both time points (56 d and 112 d), whereas the PLS loadings show six group clusters for the compounds included in the PLS-DA analysis. Figure 2 presents the expanded six cluster groups of compounds. The values in clusters 3, 5, and 6 were higher in the DHA group and in clusters 1, 4, and 2 were higher in the control group and noted with VIP scores. The clusters reveal groupings of measurements to coordinate their relationship to vital physiologic and metabolic changes in the DHA-fed mice and control mice.

Cluster 3 shows significantly higher VIP scores for the DHA group compared to the controls. Muscle values for GLUT, glucose use (AMPK, Insulin-R), and metabolism of eCB (FAAH, DAGL, NAPE-PLD, CB2 protein) coordinate with the changes in metabolites for glucose. Muscle levels in the DHA group were higher for GLUT-2 and GLUT-4, DAGL-a, FAAH, IRS1, AMPKα, Insulin-R, adenylate cyclase, and Akt-1, supporting relationships between glucose use and endocannabinoid metabolism. Further in the DHA group, higher levels of plasma OxL derived from DHA and EPA were found. In EFP, adiponectin, adenylate cyclase, GLUT-2, and NAPE-PLA revealed coordinated changes. Cluster 6 shows several significant differences in muscle and liver. Of these changes, many were related to PUFA and glycerolipids in muscle and liver. Choline, pantothenate, riboflavin, histidine, and FMN were higher in liver. Cluster 5 showed no significant increase in the DHA group.

Significant differences that were higher in the control compared to the DHA diet group including PUFA, glycerolipids, oxylipins, and glucose (liver) are shown in cluster 1. The changes in liver and muscle for PUFA, and in EFP eCB, ECS system enzymes, and proteins regulating glucose physiology and metabolism proteins are consistent with large changes in macronutrient metabolism and fat accretion in the control group. No significant differences were found based on VIP scores for biomarkers in clusters 2 and 4 (Figure 2).

## 4. Discussion

At the end of the study period, mice given the DHA semi-purified diet had less body fat, higher lean mass, and a lower body weight compared to the control diet group. However, one possible reason for no difference in body fat early on could be explained by the physiological state of growth versus maintenance at the second time point in control mice. Over the food intake collection period, no difference was found in food consumed between the DHA and control diet groups. Thus, DHA resulted in significant changes in macronutrient and systemic energy metabolism in mice leading to lower fat mass and higher lean mass at 112 d. 

Not surprisingly, feeding mice a DHA semi-purified diet led to lower levels of *n*-6 PUFA and related metabolites but higher levels of *n*-3 PUFA and DHA-derived products. AA and arachidic acid were lower in plasma of mice given the DHA diet while DHA, *n*-3 DPA, and EPA levels were higher in both liver and muscle tissue. Therefore, dietary DHA was able to modify the fatty acid composition of these tissues and various PUFA containing lipids. Our findings corroborate our previous results for mouse muscle and liver tissue fatty acid composition and plasma DHEA [10], and blood DHA and DHEA in postmenopausal women [12]. Lastly, the level of alpha-tocopherol was lower in the DHA group which may indicate that the DHA-fed mice remove more alpha-tocopherol from blood to support greater tissue accretion of *n*-3 PUFA.

Relevant to the ECS is the lowering of the AA-derived endocannabinoids, 2-AG, and 1-AG in gastrocnemius of mice fed the DHA diet in this study. The reduction in the level of these AA-derived eCB confirms the finding that *n*-3 PUFA (DHA) in myoblasts alters eCB levels [10,15]. A reduction in muscle AA-derived eCB supports the hypothesis that a DHA diet has the potential to improve ECS tone and ultimately restore control to a dysregulated modulator of systemic energy metabolism [10,11,12].

The observed changes in tissue fatty acids to DHA or to fatty acids of the DHA lineage after consuming a diet enriched in DHA highlights the potential to affect the lipid-derived ECS ligands. This is evident in the higher levels of DHA-derived glycerol lipids, 2-DHA-GPE and 1-docosahexaenoylglycerol, in muscle and liver of mice after 56 d and 112 d of consuming the DHA diet. Levels of eCB derived from AA were lower in mice, and feeding DHA to mice lowered AA and increased DHA in tissues and increased DHEA in blood [10]. Further, the treatment of DHEA in myoblast cultures was shown to increase mRNA levels of p38MAPK [15]. 

Free glucose levels in both liver and muscle were lower in the DHA diet fed mice compared to the control group. Concurrently, metabolites associated with glycolysis, such as pyruvate, 3-phosphoglycerate, and phosphoenolpyruvate, were lower in the DHA-fed mice, suggesting that the mice in the DHA group used glucose less as a fuel source, instead favoring fatty acids. The metabolite levels in the DHA group suggest reduced dependency on glucose catabolism and a shift in fuel sources to fatty acid oxidation. Lower levels of ribulose and ribose-5-phosphate in the liver, and lower flux via the pentose phosphate pathway, brings together the metabolic changes to support the observation of lower epididymal fat pad mass in the mice given the DHA diet compared to the control. Furthermore, the metabolic shift to using fat stores also corroborates the decrease in fat mass, as determined by DXA analysis [10].

In a recent review, evidence suggests that DHA likely improves glucose use by the brain [27]. The authors reported that DHA deficiency repressed GLUT-1 expression in association with low DHA levels in rat brain [27]. These findings have implications for DHA actions in the nervous system and for general neurobiological health.

Plasma levels of macronutrients strongly suggest changes in glucose and fatty acid metabolism in mice fed the DHA diet. The differences in metabolic pathway flux would support less fat accretion, improved glucose handling, and higher lean mass in mice fed the DHA diet compared to controls. Metabolites of fatty acid catabolism and glucose use associated with the Krebs cycle and glycolytic pathways, respectively, were altered in a manner consistent with the differences in body mass and composition of mice. In support of the pathway changes and fat pad mass, a reduced flux through the glycolytic pathway would result in lower αGP as found, thus resulting in lower triglyceride synthesis to explain less fat accretion observed in mice fed the DHA diet. Higher levels of related metabolites suggest greater fatty acid oxidation. A higher level of fatty acid oxidation was observed in mouse muscle as acyl-carnitines (e.g., palmitoylcarnitine, oleoylcarnitine) and acyl-glycines (e.g., hexanoylglycine and 3-hydroxybutyrate) were higher in the gastrocnemius muscle of DHA-fed mice. Thus, higher levels of activation of fatty acids suggests greater muscle oxidation of fatty acids in mice fed the DHA diet. Glucuronic acid, ethanolamine, 3-hydroxy-3-methylglutaric acid, 2-hydroxybutanoic acid, and 3-hydroxybutanoic acid were higher in the DHA group. These metabolite changes suggest greater energy expenditure or higher flux in pathways of carbohydrate use and fatty acid oxidation. Further, higher adenosine found in the DHA group of mice may support a benefit to insulin sensitivity.

In this study, mice were given one of two high-fat diets, varying in the amount of DHA but containing the same level of protein and fat. Moreover, we report lower levels of 1-arachidonylglycerol (1-AG) and 2-arachidonylglycerol (2-AG) in muscle when the DHA diet was fed, which is consistent with lower plasma 1- and 2-AG in mice fed the same diet [10].

Together these findings strengthen the relationship between the endocrine system and the ECS in characterizing obesity as a condition where the ECS is overactive and muscle glucose uptake is reduced, which in turn can lead to insulin resistance and the metabolic syndrome. These observations support our current findings of lower glucose, fat mass, and higher lean mass in mice fed the DHA diet. Further, our study is robust with dietary manipulation to alter the concentration of AA-derived and DHA-derived eCB [10]. 

A recent investigation found that DHA elevated glucose and palmitic acid oxidation in L6 rat skeletal muscle [28]. The authors suggest that DHA may increase glucose disposal and reduce lipid accumulation. These authors [28] also reported higher AMPK phosphorylation and protein levels of carnitine-palmitoyl transferase-1b (CPT1b) with DHA treatment. The findings to a great extent are consistent with Kim et al. [10,15] for glucose use and metabolism. With respect to epididymal fat pad, a higher adiponectin gene expression was reported in similar mice fed the same DHA semi-purified diet compared to those fed the control diet [10]. Adiponectin, secreted by adipocytes, increases insulin sensitivity and is associated with weight loss [29].

Among the nine amino acids that were detected, only phenylalanine and glycine were found to be higher in mice fed the DHA diet. The finding suggests that dietary DHA may act to preserve or protect specific amino acids from being catabolized in tissue while liberating others into circulation for clearance. In this regard, the plasma levels point to an overall increased utilization of amino acids in the DHA group. Creatinine was found higher in the DHA group, which may indicate a difference in energy metabolism in these mice, thus supporting energy utilization in muscle consistent with the higher lean mass in these mice. Recently, investigators have noted significant differences in amino acid levels associated with elevated glucose and decreased insulin in men [2]. 

**Figure 3:**
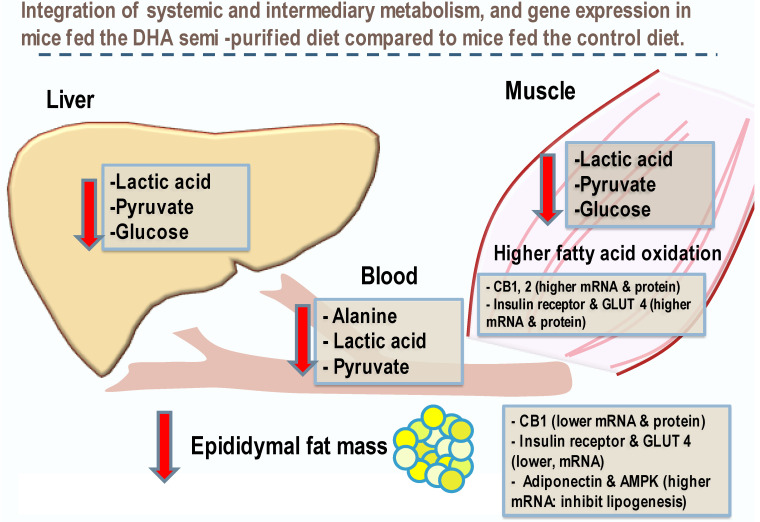
DHA feeding alters macronutrient metabolism between primary anabolic, catabolic, and energy deposition of liver, muscle, and adipose in mice. Relationships between metabolites of the glycolytic pathway and fat metabolism in systemic energy utilization were found in mice fed DHA in a semi-purified diet compared to controls fed the same semi-purified diet but with a control lipid. We make reference to previous findings for molecular aspects of glucose metabolism reported in mouse quadriceps muscle and epididymal fat pad by our laboratory (International Journal of Obesity (2016) 40, 129–137; doi:10.1038/ijo.2015.135 see article [10] for details). These findings suggest that dietary DHA reduces blood glucose and improves glucose catabolism associated with restored eCB tone when fed a high-fat diet.

To counter the overactivation of the ECS, dietary DHA appears to improve ECS tone and support better clearance of glucose from blood by increasing glucose transporters to the sarcolemma of the muscle [10]. Incorporating DHA into the diet of an obese individual may have the potential of restoring balance and reverse the dysregulation of the ECS, resulting in an increase of glucose uptake into skeletal muscle and a reduction in insulin resistant-associated hyperglycemia. Our findings on systemic energy metabolism including glucose are summarized in Figure 3.

## 5. Conclusions

Collectively, our findings demonstrate that a DHA diet fed to mice resulted in a lower glucose circulating level, greater flux through pathways of macronutrient metabolism, and higher lipid catabolism compared to controls. The metabolite changes support the finding of lower fat pad mass and higher lean mass in DHA-fed mice. DHA feeding to mice suggests a restored eCB tone, resulting in lower fat mass, greater lean mass, and lowered AA-derived eCB in muscle. A role for dietary DHA is emerging to control blood glucose and facilitate changes in pathways of intermediary metabolism in muscle and liver, and systemic handling of macronutrients.

## Figures and Tables

**Table 1:** Plasma amino acids and their degradation products in mice after 56 d of being fed the semi-purified diets.

	Control		DHA		*t*-Test *p* Value
	Mean	SD	Mean	SD	
**Amino acids**					
tyrosine	85,852	38,539	52,368	11,774	0.033
tryptophan	72,909	11,537	63,447	4194	0.043
threonine	41,289	7621	29,157	7198	0.0031
serine	41,423	10,518	27,102	8210	0.0053
proline	52,514	40,615	20,335	7564	0.046
phenylalanine	11,010	2585	13,487	1602	0.027
glycine	106,926	15,281	134,448	24,771	0.012
aspartic acid	1620	563	860	218	0.0034
alanine	223,360	69,824	138,634	45,823	0.0077
**Catabolites of amino acids**					
N-methylalanine	2137	491	8811	3828	0.0008
N-hexanoylglycine	152	30	426	216	0.0052
cystine	1297	224	2274	431	<0.0001
creatinine	9344	2905	19,442	3232	<0.0001
4-hydroxyproline	3088	480	2497	521	0.024

N = 9 for both groups. Data were analyzed by Student’s *t*-test using SAS 9.4 software. Data in the table were relative quantitation values that were normalized to the height of the compound’s peak, which made these values unitless.

**Table 2:** Plasma fatty acids, carbohydrates, and alcohols in mice after 56 d of being fed the semi-purified diets.

	Control		DHA		*t*-Test *p* Value
	Mean	SD	Mean	SD	
**Fatty acids**					
palmitoleic acid	4616	2445	9322	3450	0.0042
palmitic acid	29,849	6148	39,137	6262	0.0059
oleic acid	3141	1258	6293	2218	0.0019
linoleic acid	3675	1282	7538	2362	0.0005
lauric acid	1289	216	2686	857	0.0011
eicosenoic acid	562	241	790	216	0.049
arachidonic acid	4891	1573	1186	221	<0.0001
arachidic acid	2103	661	1583	183	0.048
**Carbohydrates and alcohols**					
maltose	367	185	155	78	0.0093
levanbiose	962	468	378	169	0.0055
fucose	948	158	1147	213	0.034
fructose	13,382	10,015	4847	1994	0.035

N = 9 for both groups. Data were analyzed by Student’s *t*-test using SAS 9.4 software. Data in the table were relative quantitation values that were normalized to the height of the compound’s peak, which made these values unitless.

**Table 3:** Plasma metabolites of macronutrient catabolism in mice after 56 d of being fed the semi-purified diets.

	Control		DHA		*t*-Test *p* Value
	Mean	SD	Mean	SD	
**Metabolites**					
pyruvic acid	16,273	5392	8886	4338	0.0056
malic acid	5116	1898	3281	1170	0.025
lactic acid	323,139	50,103	181,218	38,740	<0.0001
alpha-glycerol phosphate	5315	1178	3857	969	0.011
glucuronic acid	1153	298	1737	255	0.0004
ethanolamine	3349	702	5089	358	<0.0001
3-hydroxybutanoic acid	14,341	4179	254,100	143,147	0.001
3-hydroxy-3-methylglutaric acid	75	16	261	138	0.0036
2-hydroxybutanoic acid	4660	971	38,966	29,245	0.0078

N = 9 for both groups. Data were analyzed by Student’s *t*-test using SAS 9.4 software. Data in the table were relative quantitation values that were normalized to the height of the compound’s peak, which made these values unitless.

**Table 4:** Plasma nucleotides, vitamin E, and pyrophosphate in mice after 56 d of being fed the semi-purified diets.

	Control		DHA		*t*-Test *p* Value
	Mean	SD	Mean	SD	
**Nucleotides**					
uridine	1622	423	2096	334	0.018
uracil	1728	351	2241	493	0.022
thymine	614	175	918	226	0.0057
thymidine	962	256	1342	303	0.011
adenosine-5-phosphate	363	625	2133	1726	0.016
adenosine	211	225	777	672	0.038
**Vitamin**					
Alpha-tocopherol	3609	995	2292	393	0.0039
**Inorganic compound**					
pyrophosphate	758	139	2340	1269	0.0056

N = 9 for both groups. Data were analyzed by Student’s *t*-test using SAS 9.4 software. Data in the table were relative quantitation values that were normalized to the height of the compound’s peak, which made these values unitless.

**Table 5:** Levels of PUFA in liver and muscle of mice after 56 d and 112 d of being fed the semi-purified diets.

	Control		DHA		*p* Value
	Mean	SD	Mean	SD	
**56 d**					
**Liver**					
arachidonate (20:4*n*-6)	1.3200	0.2098	0.7471	0.1867	0.0011
eicosapentaenoate (EPA; 20:5*n*-3)	0.0383	0.0095	4.3093	1.8281	<0.0001
adrenate (22:4*n*-6)	1.6180	0.3126	0.4425	0.1733	<0.0001
docosapentaenoate (*n*-6 DPA; 22:5*n*-6)	2.7606	0.5911	0.0759	0.0546	2.7606
docosapentaenoate (*n*-3 DPA; 22:5*n*-3)	0.1651	0.0534	4.8068	2.3997	0.1651
docosahexaenoate (DHA; 22:6*n*-3)	0.1332	0.0313	3.7255	1.1794	0.1332
**Muscle**					
arachidonate (20:4*n*-6)	1.8291	0.3045	0.3819	0.0608	<0.0001
eicosapentaenoate (EPA; 20:5*n*-3)	0.2834	0.0721	3.3461	1.0881	<0.0001
adrenate (22:4*n*-6)	1.4705	0.3384	0.2057	0.0884	<0.0001
docosapentaenoate (*n*-6 DPA; 22:5*n*-6)	2.4973	0.4189	0.0275	0.0071	<0.0001
docosapentaenoate (*n*-3 DPA; 22:5*n*-3)	0.4415	0.1253	2.3727	0.6880	<0.0001
docosahexaenoate (DHA; 22:6*n*-3)	0.2299	0.0416	2.7188	0.5966	<0.0001
**112 d**					
**Liver**					
arachidonate (20:4*n*-6)	1.3756 *	0.4703	0.7058 *	0.1775	0.0007
eicosapentaenoate (EPA; 20:5*n*-3)	0.0468	0.0242	3.7419 *	1.0048	<0.0001
adrenate (22:4*n*-6)	2.6503	2.4065	0.5032 *	0.2750	0.0004
docosapentaenoate (*n*-6 DPA; 22:5*n*-6)	1.3666	0.8968	0.0207 *	0.0000	<0.0001
docosapentaenoate (*n*-3 DPA; 22:5*n*-3)	0.1165 *	0.0742	3.3629	1.4651	<0.0001
docosahexaenoate (DHA; 22:6*n*-3)	0.1302	0.0624	2.4897 *	0.4070	<0.0001
**Muscle**					
arachidonate (20:4*n*-6)	1.8341 *	0.4598	0.4217 *	0.1501	<0.0001
eicosapentaenoate (EPA; 20:5*n*-3)	0.0700 *	0.0024	1.5265 *	0.6380	<0.0001
adrenate (22:4*n*-6)	1.5253 *	0.4541	0.4994	0.1958	<0.0001
docosapentaenoate (*n*-6 DPA; 22:5*n*-6)	1.8794 *	0.4413	0.0463	0.0154	<0.0001
docosapentaenoate (*n*-3 DPA; 22:5*n*-3)	0.2959 *	0.0906	2.5474	1.1073	<0.0001
docosahexaenoate (DHA; 22:6*n*-3)	0.1480 *	0.0466	3.7247	1.5592	<0.0001

N = 9 for both groups. Data were analyzed by Welch’s two-sample *t*-test with the program “R.” Data in the table were relative quantitation values that were rescaled from raw area counts for each metabolite by dividing all sample values by the median value for each individual metabolite. These values are unitless. *, values are different between 56 d and 112 d by Welch’s two-sample *t*-test.

**Table 6:** Levels of glycerolipids in liver and muscle of mice after 56 d and 112 d of being fed the semi-purified diets.

Biochemical Name	Control		DHA		*p* Value
	Mean	SD	Mean	SD	
**56 d**					
**Liver**					
2-arachidonoylglycerophosphocholine	2.7940	1.2625	0.5608	0.3476	0.0030
2-docosapentaenoylglycerophosphoethanolamine	4.2330	1.9818	0.7497	0.1830	0.0001
2-docosahexaenoylglycerophosphoethanolamine	0.7170	0.2639	4.4960	3.3225	0.0012
2-eicosapentaenoylglycerophosphoethanolamine	0.3616	0.0207	2.7737	2.5555	0.0080
1-arachidonoylglycerophosphocholine	2.4145	0.9349	0.4920	0.2636	<0.0001
**Muscle**					
2-docosapentaenoylglycerophosphoethanolamine	3.0663	0.9060	0.1189	0.0181	<0.0001
1-arachidonoylglycerophosphoethanolamine	1.4516	0.3785	0.3563	0.1214	<0.0001
2-arachidonoylglycerophosphoethanolamine	2.0655	0.5595	0.6371	0.1363	<0.0001
2-arachidonoylglycerophosphocholine	2.5880	0.8560	0.6273	0.1845	<0.0001
2-docosapentaenoylglycerophosphocholine	3.6155	1.2326	0.1808	0.0461	<0.0001
2-docosahexaenoylglycerophosphocholine	0.3044	0.0943	1.8794	0.3765	<0.0001
2-docosahexaenoylglycerophosphoethanolamine	0.4929	0.1213	1.5503	0.2128	<0.0001
1-arachidonoylglycerophosphoinositol	1.6947	0.7037	0.8411	0.1872	0.0033
2-arachidonoylglycerophosphoinositol	1.6819	0.8065	0.6803	0.2287	0.0112
1-docosahexaenoylglycerol (1-monodocosahexaenoin)	0.3779	0.1648	2.2747	0.7358	<0.0001
**112 d**					
**Liver**					
2-arachidonoylglycerophosphocholine	1.3106	1.1361	3.4877 *	3.2535	0.1489
2-docosapentaenoylglycerophosphoethanolamine	Not in 4 months				
2-docosahexaenoylglycerophosphoethanolamine	0.2310	0.0866	3.9030	3.2447	0.0001
2-eicosapentaenoylglycerophosphoethanolamine	0.2742	0.0000	0.9972	0.8110	0.0268
1-arachidonoylglycerophosphocholine	1.7012	0.7104	0.5098	0.3089	0.0003
**Muscle**					
2-docosapentaenoylglycerophosphoethanolamine	2.7814	1.6277	0.4282 *	0.3144	<0.0001
1-arachidonoylglycerophosphoethanolamine	1.4671 *	0.4190	0.5526	0.2670	0.0001
2-arachidonoylglycerophosphoethanolamine	1.0922 *	0.2348	0.9295 *	0.1613	<0.0001
2-arachidonoylglycerophosphocholine	1.8327	1.3455	0.6788	0.2858	0.0396
2-docosapentaenoylglycerophosphocholine	1.8583	1.3056	0.4568	0.1295	0.0023
2-docosahexaenoylglycerophosphocholine	0.4360	0.0000	1.3770	0.6039	0.0003
2-docosahexaenoylglycerophosphoethanolamine	0.6673	0.2887	4.3251 *	2.0894	<0.0001
1-arachidonoylglycerophosphoinositol	1.1573 *	0.3486	0.6923 *	0.2872	0.0101
2-arachidonoylglycerophosphoinositol	1.0242 *	0.5939	0.9133	0.6687	0.4251
1-docosahexaenoylglycerol (1-monodocosahexaenoin)	0.2058	0.0000	1.7064	0.8264	<0.0001

N = 9 for both groups. Data were analyzed by Welch’s two-sample *t*-test with the program “R”. Data in the table were relative quantitation values that were rescaled from raw area counts for each metabolite by dividing all sample values by the median value for each individual metabolite. These values are unitless. *, values are different between 56 d and 112 d by Welch’s two-sample *t*-test.

**Table 7:** Levels of endocannabinoids and related compounds in liver and muscle of mice after 56 d and 112 d of being fed the semi-purified diets.

	Control		DHA		*p* Value
	Mean	SD	Mean	SD	
**56 d**					
**Liver**					
palmitoyl ethanolamide	0.8058	0.1166	1.6410	0.5305	0.0017
N-palmitoyl taurine	0.1327	0.0593	2.5040	1.4256	<0.0001
oleoyltaurine	0.4283	0.1164	5.1876	5.0201	0.0004
**Muscle**					
1-arachidonylglycerol (1-AG)	2.4248	1.0427	0.4333	0.1549	<0.0001
2-arachidonoylglycerol (2-AG)	1.7126	0.4429	0.3889	0.1781	<0.0001
palmitoyl ethanolamide	0.9580	0.2682	2.4146	1.7986	0.0375
oleic ethanolamide	0.9188	0.2264	2.1369	1.5665	0.0634
**112 d**					
**Liver**					
palmitoyl ethanolamide	0.6470	0.2429	1.3496	0.4241	0.0019
N-palmitoyl taurine	0.1082	0.0228	0.9029	0.4263	0.0001
oleoyltaurine	0.2170	0.1511	1.2880	1.1608	0.0046
**Muscle**					
1-arachidonylglycerol	Not at 112 d				
2-arachidonoylglycerol	Not at 112 d				
palmitoyl ethanolamide	1.0270	0.3415	0.9839	0.3476	0.7891
oleic ethanolamide	0.8023	0.3608	0.7688 *	0.2992	0.8922

N = 9 for both groups. Data were analyzed by Welch’s two-sample *t*-test with the program “R”. Data in the table were relative quantitation values that were rescaled from raw area counts for each metabolite by dividing all sample values by the median value for each individual metabolite. These values are unitless. *, values are different between 56 d and 112 d by Welch’s two-sample *t*-test.

**Table 8:** Levels of fatty acid oxidation and catabolism products in liver and muscle of mice after 56 d and 112 d of being fed the semi-purified diets.

Biochemical Name	Control		DHA		*p* Value
	Mean	SD	Mean	SD	
**56 d**					
**Liver**					
3-hydroxybutyrate (BHBA)	0.6381	0.1356	3.8559	1.9848	<0.0001
**Muscle**					
palmitoylcarnitine	0.6851	0.3402	1.7158	0.8171	0.0053
oleoylcarnitine	0.6307	0.3216	1.7390	0.8521	0.0032
hexanoylglycine	0.3882	0.1902	15.7466	18.7037	0.0007
3-hydroxybutyryl CoA	0.7137	0.1537	1.3123	0.6923	0.0277
3-hydroxybutyrate (BHBA)	0.5473	0.1536	5.2007	3.0272	<0.0001
adenosine 5′-monophosphate (AMP)	1.1277	0.1650	0.7741	0.4061	0.0169
adenosine 5′-diphosphate (ADP)	0.6917	0.2505	1.5284	0.4130	0.0008
**112 d**					
**Liver**					
3-hydroxybutyrate (BHBA)	0.8843	0.2934	1.7060 *	0.8838	0.0074
**Muscle**					
palmitoylcarnitine	2.8918 *	3.1653	3.4229 *	2.7655	0.5528
oleoylcarnitine	2.8381 *	3.2974	3.9475 *	3.5068	0.4254
hexanoylglycine	0.5422	0.3601	1.1493 *	0.8596	0.0826
3-hydroxybutyryl CoA	0.7672	0.2954	1.4205	1.1271	0.2040
3-hydroxybutyrate (BHBA)	0.8988	0.3041	1.8699 *	1.1540	0.0234
adenosine 5′-monophosphate (AMP)	1.1852 *	0.4944	1.2063 *	0.4636	0.9340
adenosine 5′-diphosphate (ADP)	0.9868	0.3402	1.1514	0.5474	0.5672

N = 9 for both groups. Data were analyzed by Welch’s two-sample *t*-test with the program “R.” Data in the table were relative quantitation values that were rescaled from raw area counts for each metabolite by dividing all sample values by the median value for each individual metabolite. These values are unitless. *, values are different between 56 d and 112 d by Welch’s two-sample *t*-test.

**Table 9:** Levels of glucose and other related metabolites in liver and muscle of mice after 56 d and 112 d of being fed the semi-purified diets.

Biochemical Name	Control		DHA		*p* Value
	Mean	SD	Mean	SD	
**56 d**					
**Liver**					
maltose	2.0175	0.8141	0.0038	0.0016	<0.0001
maltotriose	1.3509	0.8221	0.0004	0.0001	<0.0001
maltopentaose	1.0436	0.7799	0.0021	0.0000	0.0001
maltohexaose	1.0157	0.8885	0.0153	0.0000	0.0042
maltotetraose	0.8376	0.5276	0.0221	0.0000	0.0002
pyruvate	2.5548	0.8936	0.6497	0.2189	<0.0001
lactate	1.3384	0.1323	0.4734	0.1707	<0.0001
glucose	1.7837	0.3188	0.4002	0.2106	<0.0001
Isobar: fructose 1,6-diphosphate, glucose 1,6-diphosphate, myo-inositol 1,4 or 1,3-diphosphate	4.5362	4.5623	0.6865	0.1862	0.0127
2-phosphoglycerate	0.8240	0.0956	2.5755	0.9130	0.0002
ribose 5-phosphate	2.2305	0.9766	0.4596	0.1719	<0.0001
ribulose	2.3756	0.6243	0.2295	0.0989	<0.0001
**Muscle**					
pyruvate	1.6086	0.5613	0.7317	0.1548	0.0001
maltose	1.6501	0.7741	0.8315	0.4711	0.0155
maltotriose	2.0827	0.9314	0.5998	0.5298	0.0003
maltotetraose	2.1516	0.8210	0.6948	0.2313	<0.0001
lactate	1.1204	0.0794	0.9282	0.0622	0.0001
glucose	1.5782	0.5255	0.5757	0.1915	<0.0001
Isobar: fructose 1,6-diphosphate, glucose 1,6-diphosphate, myo-inositol 1,4 or 1,3-diphosphate	8.3932	10.4943	0.5177	0.2071	0.0008
3-phosphoglycerate	3.0852	1.4674	0.2206	0.1527	<0.0001
phosphoenolpyruvate (PEP)	3.1129	1.4451	0.3315	0.1862	<0.0001
2-phosphoglycerate	3.9703	2.1867	0.2620	0.1473	<0.0001
**112 d**					
**Liver**					
maltose	1.5666	0.3057	0.1084 *	0.2613	<0.0001
maltotriose	0.9200	0.4206	0.0058	0.0141	<0.0001
maltopentaose	1.8318	1.6903	0.3734	0.0000	0.0060
maltohexaose	2.8602	3.2058	0.1328	0.0000	0.0024
maltotetraose	8.1343	3.8914	0.0669	0.1340	<0.0001
pyruvate	1.4437 *	0.5622	0.6509	0.2122	0.0002
lactate	1.0923 *	0.0839	0.7151 *	0.2151	0.0028
glucose	1.2194 *	0.1190	0.6478 *	0.2034	0.0003
Isobar: fructose 1,6-diphosphate, glucose 1,6-diphosphate, myo-inositol 1,4 or 1,3-diphosphate	Not in 4 months				
2-phosphoglycerate	1.2386	0.6371	0.7704 *	0.4124	0.0682
ribose 5-phosphate	1.4359	0.6230	0.2070	0.0503	<0.0001
ribulose	1.4699 *	0.3598	0.7230 *	0.3396	0.0030
**Muscle**					
pyruvate	1.0608 *	0.3120	1.2920	0.6198	0.5590
maltose	1.0113 *	0.2436	1.0024	0.3229	0.8228
maltotriose	1.1744	0.4072	1.2715 *	0.5187	0.7942
maltotetraose	1.1476 *	0.2700	1.2501 *	0.4376	0.6991
lactate	0.9053 *	0.1892	0.8895 *	0.1713	0.8818
glucose	0.9621 *	0.2857	0.8596	0.3114	0.4229
Isobar: fructose 1,6-diphosphate, glucose 1,6-diphosphate, myo-inositol 1,4 or 1,3-diphosphate	1.6254 *	1.0197	2.3219 *	2.0926	0.6949
3-phosphoglycerate	1.3271 *	0.7579	2.0236 *	1.7252	0.7639
phosphoenolpyruvate (PEP)	1.2280 *	0.7003	1.9336 *	1.6093	0.6383
2-phosphoglycerate	1.3441 *	0.7689	1.9184 *	1.6803	0.9132

N = 9 for both groups. Data were analyzed by Welch’s two-sample *t*-test with the program “R.” Data in the table were relative quantitation values that were rescaled from raw area counts for each metabolite by dividing all sample values by the median value for each individual metabolite. These values are unitless. *, values are different between 56 d and 112 d by Welch’s two-sample *t*-test.

## Data Availability

Available upon request.

## Supplementary Materials

The following supporting information can be downloaded at: https://www.mdpi.com/article/10.3390/nu15122679/s1, Table S1: Ingredient composition and major fatty acids in the semi-purified diets fed to mice; Table S2: Mouse body weights and whole-body dual-energy X-ray absorptiometry (DXA) data at 56 and 112 d; Figure S1: Amino acids and their metabolites of the glycolytic and the Krebs cycle pathways; Figure S2: Degradation of amino acids, energy balance, and nucleic acids; Figure S3: Metabolites of fatty acid and glucose degradation associated with the glycolytic and the Krebs cycle pathways; Figure S4: Ethanolamine metabolism; Figure S5: Catabolism of nucleotides.

## Abbreviations

1-mono DHA	1-docosahexaenoylglycerol
1-AG	1-arachidonylglycerol
2-AG	2-arachidonylglycerol
2-DHA-GPE	2-docosahexaenoylglycerophosphoethanolamine
9-HODE	9-hydroxy-10,12-octadedadienoic acid
12-HETE	12-hydroxyeicosatetraenoic acid
13-HODE	13-hydroxy-9,11-octadecadienoic acid
AA	arachidonic acid
AEA	N-arachidonoylethanolamine; anandamide
AMP	adenosine 5’-monophosphate
ADP	adenosine 5’-diphosphate
BHBA	3-hydroxybutyrate
CB1	cannabinoid receptor 1
CB2	cannabinoid receptor 2
DHA	docosahexaenoic acid
DHEA	docosahexaenoyl ethanolamide
eCB	endocannabinoid
ECS	endocannabinoid system
EPA	eicosapentaenoic acid
FAME	fatty acid methyl ester
MAPK	mitogen-activated protein kinase
PUFA	polyunsaturated fatty acid
