# Supplementary material for: Dietary Docosahexaenoic Acid and Glucose Systemic Metabolic Changes in the Mouse
Source: Nutrients. 2023 Jun 8;15(12):2679. doi: 10.3390/nu15122679 (PMC10303726; doi:10.3390/nu15122679)
Supplement: Supplementary file 1 [file nutrients-15-02679-s001.zip › nutrients-2404189-supplementary.pdf]

Supplemental Materials

Table S1. Ingredient composition and major fatty acids in the semi-purified diets fed to mice

Fatty acid (wt%)	Dietary treatments[a,b,c,d]	
	Control	DHA
**Saturated**	9.7	11.7
12:0,14:0, 16:0, 18:0, 20:0, 22:0		
**Monounsaturated**	16.8	18.5
16:1n-7, 18:1n-9 22:1n-9, 24:1n-9		
**Polyunsaturated**		
18:2n-6	72.9	63.6
18:3n-3	0.2	0.1
22:6n-3	0.0	5.9
**Polyunsaturated totals**	73.1	69.7
n-6 PUFA	72.9	63.6
n-3 PUFA	0.2	6.0
**Ratio of n-6:n-3**	>300	10.5

[a]The semipurified basal diet contains the following (g/kg): casein, 200; corn starch, 367.076; DYETROSE, 122; sucrose, 100; cellulose, 50; L-lysine, 3; choline bitartrate, 2.5; salt mix, 35; vitamin mix, 10 (based on Watkins et al, 2006).

[b]Salt mix provides (mg/kg diet): $CaCO_3$, 12495; K_2HPO_4, 6860; $C_6H_5O_7K_3 \cdot H_2O$, 2477; NaCl, 2590; K_2SO_4, 1631; MgO, 840; $C_6H_5O_7Fe$, U.S.P., 212.1; $ZnCO_3$, 57.75; $MnCO_3$, 22.05; $CuCO_3$, 10.5; KIO_3, 0.35; Na_2SeO_4, 0.359; $(NH_4)_2MoO_4 \cdot H_2O$, 0.278; $Na_2O_3Si \cdot 9H_2O$, 50.75; $CrK(SO_4)_2 \cdot 12H_2O$, 9.625; LiCl, 0.609; H_3BO_3, 2.853; NaF, 2.223; $NiCO_3$, 1.113; NH_4VO_3, 0.231.

[c]Vitamin mix provides (mg/kg diet): thiamine HCl, 6; riboflavin, 6; pyridoxine HCl, 17; niacin, 30; calcium pantothenate, 16; folic acid, 2; biotin, 0.2; cyanocobalamin (B_{12}) (0.1%), 25; vitamin A palmitate (500,000 IU/g), 8; vitamin E acetate (500 IU/g), 150; vitamin D_3, 2.5; vitamin K_1, 0.75.

[d]Dietary fat treatments includes safflower oil (Control), 86.8% safflower oil + 13.2% DHASCO oil (DHA). Total fat content in each diet is 110.4 g/kg of diet.

PUFA, total n-6 and n-3 polyunsaturated fatty acids.

Values for fatty acids are the analysis of 6 samples of dietary lipid in final diet mixture.

Table S2. Mouse body weights and whole-body dual-energy X-ray absorptiometry (DXA) data at 56 and 112 d.

56 days DXA analysis

Variable	Control		DHA		t-test p value
	Mean	SD	Mean	SD	
Body weight (g)	31.1	3.2	28.1	1.7	0.025
BMD (g/cm^2)	0.065	0.006	0.062	0.002	0.12
BMC (g)	0.756	0.132	0.766	0.106	0.86
Lean mass (g)	16.6	3.00	15.9	3.42	0.64
Fat mass (g)	11.6	5.6	9.28	3.66	0.32
BMC/BW (mg/g)	24.2	2.47	27.2	3.30	0.043
Lean/BW (g/g)	0.55	0.14	0.57	0.13	0.74
Fat/BW (g/g)	0.36	0.14	0.33	0.13	0.64

112 days DXA analysis

Variable	Control		DHA		t-test p value
	Mean	SD	Mean	SD	
Body weight (g)	39.7	4.0	35.7	3.8	0.044
BMD (g/cm^2)	0.066	0.006	0.068	0.002	0.51
BMC (g)	1.033	0.183	0.943	0.142	0.27
Lean mass (g)	12.56	4.41	17.59	2.55	0.001
Fat mass (g)	23.79	6.75	14.61	5.05	0.001
BMC/BW (mg/g)	26.0	3.9	24.2	7.4	0.52
Lean/BW (g/g)	0.32	0.13	0.50	0.10	0.005
Fat/BW (g/g)	0.59	0.14	0.40	0.11	0.005

Determinations for body composition performed by dual-energy X-ray absorptiometry (DXA). N = 9 for both groups at both time points. SD = standard deviation.

Figures S1–S5. Changes in macronutrient metabolism in mice fed the DHA-diet compared to the control semi-purified diet group.

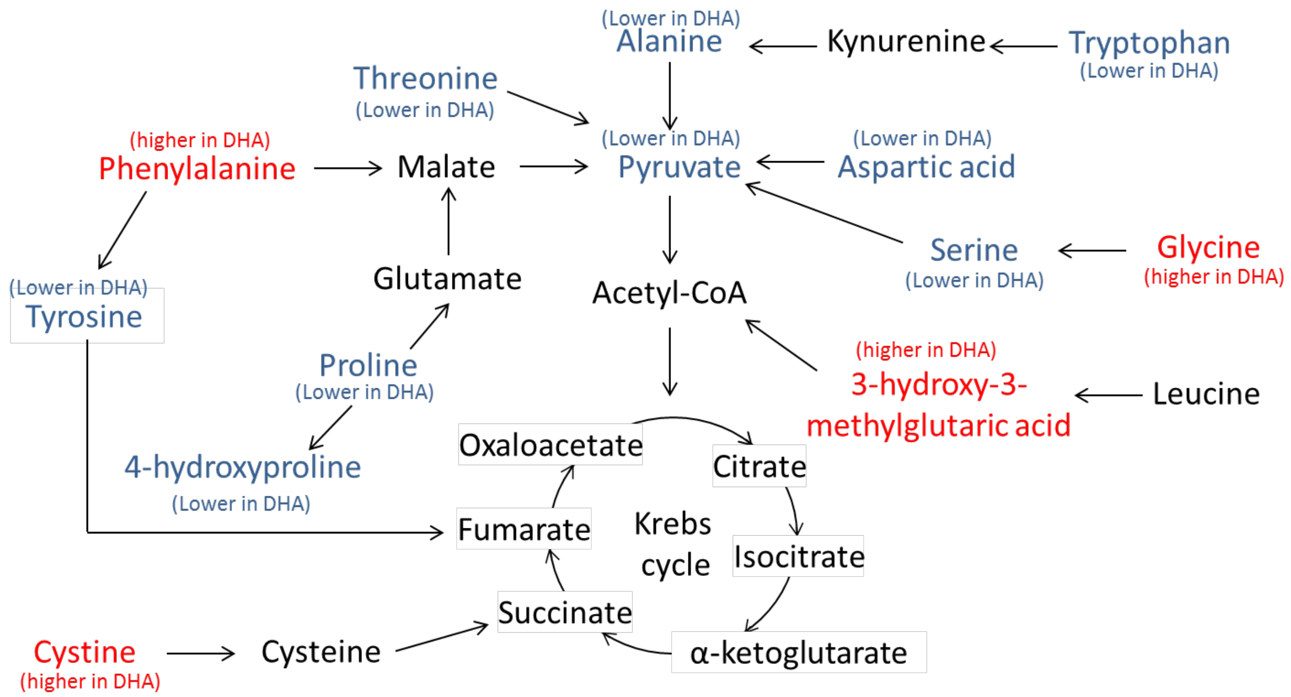

Figure S1. Amino acids and their metabolites of the glycolytic and the Krebs cycle pathways.

Note that compounds in red font were higher while those in blue font were lower for the mice fed the DHA semi-purified diet compared to those values in mice fed the control diet.

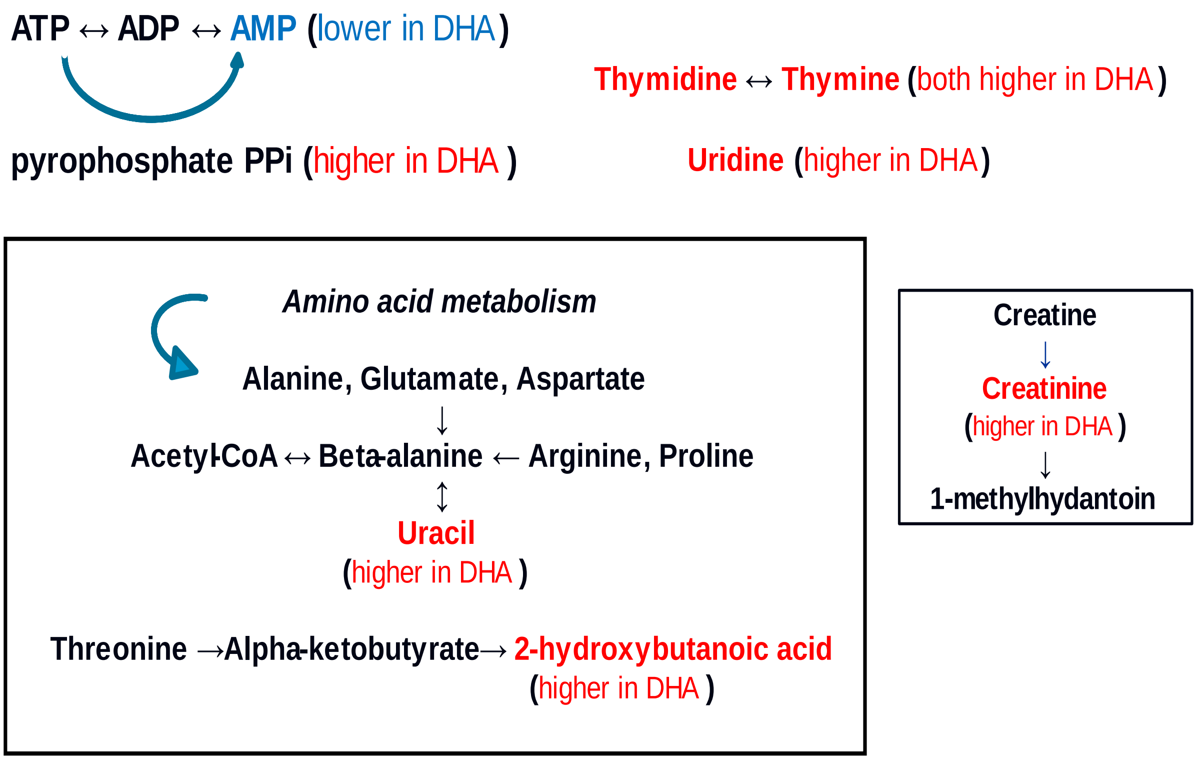

Figure S2. Degradation of amino acids, energy balance, and nucleic acids.

Levels of adenosine triphosphate (ATP), adenosine 5'-diphosphate (ADP), adenosine 5'-monophosphate (AMP), and pyrophosphate were affected by DHA feeding in plasma, liver, and muscle of mice. AMP and ADP, which are lower and higher, respectively, in muscle at 56 d for the DHA group compared to the control group (Table 8 of text), however, AMP level was higher over time at 112 d. In contrast, plasma adenosine-5-phosphate was higher in the mice fed the DHA-diet (Table 4 of text). Note that compounds in red font were higher while those in blue font were lower for the mice fed the DHA semi-purified diet compared to those values in mice fed the control diet.

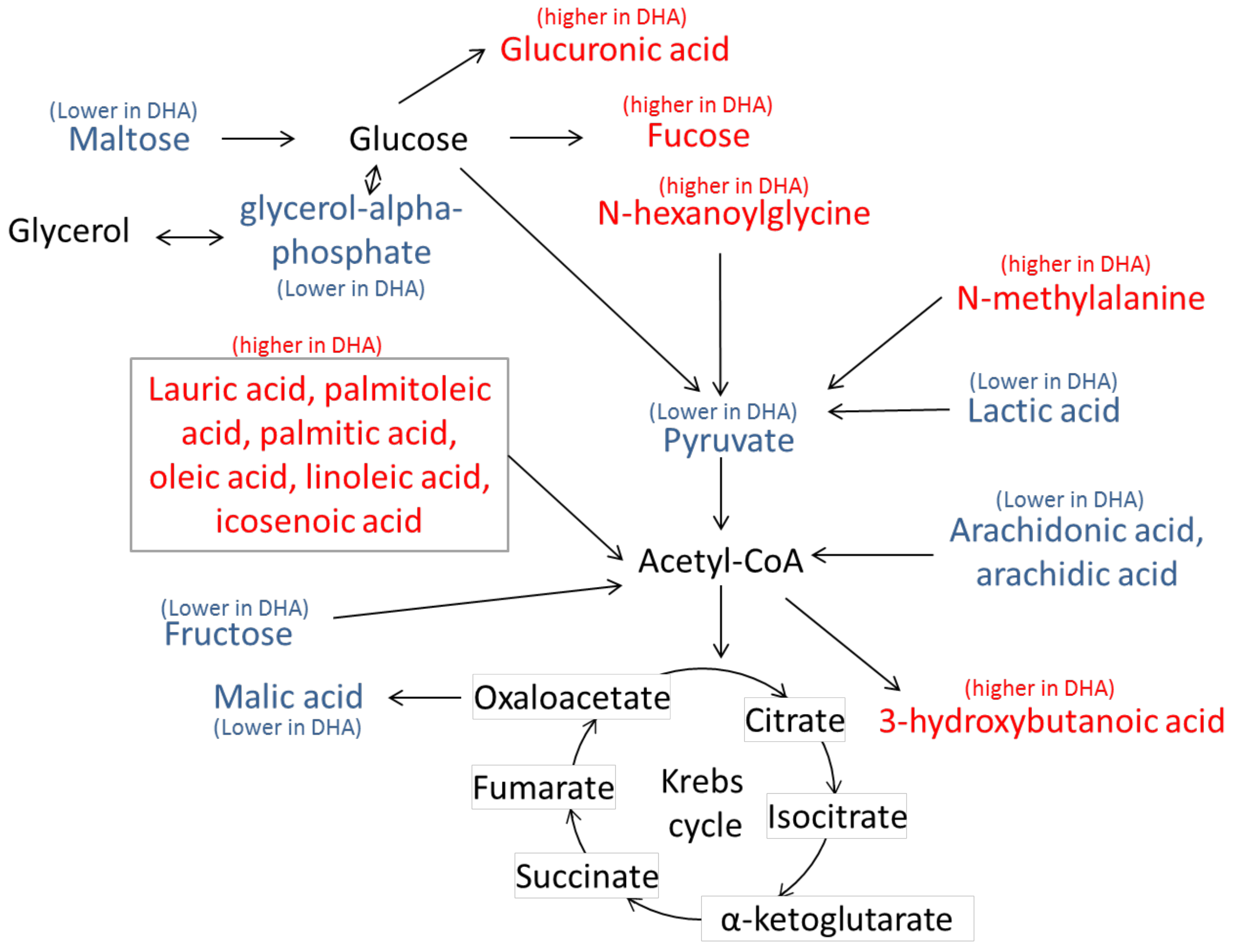

Figure S3. Metabolites of fatty acid and glucose degradation associated with the glycolytic and the Krebs cycle pathways.

Note that compounds in red font were higher while those in blue font were lower for the mice fed the DHA semi-purified diet compared to those values in mice fed the control diet.

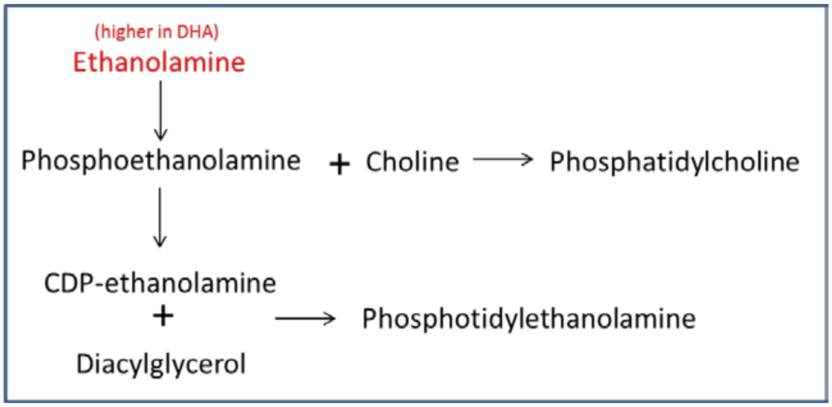

Figure S4. Ethanolamine metabolism.

Note that compounds in red font were higher while those in blue font were lower for the mice fed the DHA semi-purified diet compared to those values in mice fed the control diet.

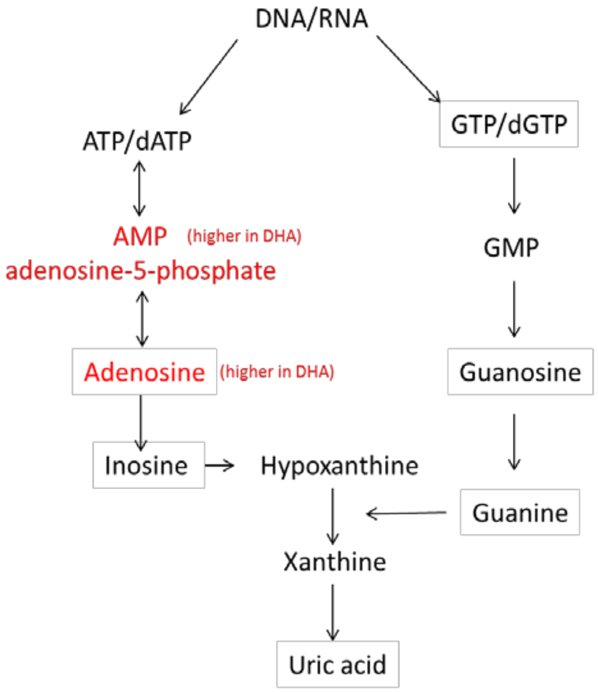

Figure S5. Catabolism of nucleotides.

AMP and adenosine values are from plasma (refer to Table 4 of text). Note that compounds in red font were higher while those in blue font were lower for the mice fed the DHA semi-purified diet compared to those values in mice fed the control diet.
